# Desire and reality – teaching and assessing communicative competencies in undergraduate medical education in German-speaking Europe – a survey

**DOI:** 10.3205/zma000998

**Published:** 2015-11-16

**Authors:** Anja Härtl, Cadja Bachmann, Katharina Blum, Stefan Höfer, Tim Peters, Ingrid Preusche, Bianca Raski, Stefan Rüttermann, Michaela Wagner-Menghin, Alexander Wünsch, Claudia Kiessling, GMA-Ausschuss Kommunikative und Soziale Kompetenzen

**Affiliations:** 1 Klinikum der Universität München, Institut für Didaktik und Ausbildungsforschung in der Medizin, München, Deutschland; 2 Universitätsklinikum Hamburg-Eppendorf, Institut für Allgemeinmedizin, Hamburg, Deutschland; 3 Medizin Universität Innsbruck, Universitätsklinik für Medizinische Psychologie, Innsbruck, Österreich; 4 Ruhr-Universität Bochum, Medizinische Fakultät, Zentrum für Medizinische Lehre, Bochum, Deutschland; 5 Medizinische Universität Wien, Department für Medizinische Aus- und Weiterbildung, Wien, Österreich; 6 Universitätsklinikum Düsseldorf, Studiendekanat, Düsseldorf, Deutschland; 7 Universitätsklinikum Düsseldorf, Klinisches Institut für Psychosomatische Medizin und Psychotherapie, Düsseldorf, Deutschland; 8 Goethe-Universität Frankfurt, ZZMK Carolinum, Poliklinik für Zahnerhaltung, Frankfurt, Deutschland; 9 Klinikum rechts der Isar, Klinik und Poliklinik für Psychosomatische Medizin und Psychotherapie, München, Deutschland; 10 Technische Universität München, TUM MeDiCAL, München, Deutschland; 11 Medizinische Hochschule Brandenburg Theodor Fontane, Bereich Assessment und Prüfungsorganisation, Neuruppin, Deutschland

**Keywords:** medical studies, communicative competencies, instruction, assessment, longitudinal curriculum

## Abstract

**Objectives: **Increasingly, communicative competencies are becoming a permanent feature of training and assessment in German-speaking medical schools (n=43; Germany, Austria, Switzerland – ”D-A-CH”). In support of further curricular development of communicative competencies, the survey by the “Communicative and Social Competencies” (KusK) committee of the German Society for Medical Education (GMA) systematically appraises the scope of and form in which teaching and assessment take place.

**Methods: **The iterative online questionnaire, developed in cooperation with KusK, comprises 70 questions regarding instruction (n=14), assessment (n=48), local conditions (n=5), with three fields for further remarks. Per location, two to three individuals who were familiar with the respective institute’s curriculum were invited to take part in the survey.

**Results:** Thirty-nine medical schools (40 degree programmes) took part in the survey. Communicative competencies are taught in all of the programmes. Ten degree programmes have a longitudinal curriculum for communicative competencies; 25 programmes offer this in part. Sixteen of the 40 programmes use the Basler Consensus Statement for orientation. In over 80% of the degree programmes, communicative competencies are taught in the second and third year of studies. Almost all of the programmes work with simulated patients (n=38) and feedback (n=37). Exams are exclusively summative (n=11), exclusively formative (n=3), or both summative and formative (n=16) and usually take place in the fifth or sixth year of studies (n=22 and n=20). Apart from written examinations (n=15) and presentations (n=9), practical examinations are primarily administered (OSCE, n=31); WPA (n=8), usually with self-developed scales (OSCE, n=19). With regards to the examiners’ training and the manner of results-reporting to the students, there is a high variance.

**Conclusions:** Instruction in communicative competencies has been implemented at all 39 of the participating medical schools. For the most part, communicative competencies instruction in the D-A-C-H region takes place in small groups and is tested using the OSCE. The challenges for further curricular development lie in the expansion of feedback, the critical evaluation of appropriate assessment strategies, and in the quality assurance of exams.

## Zusammenfassung

**Zielsetzung:** An deutschsprachigen medizinischen Fakultäten (n=43, Deutschland, Österreich, Schweiz; (D-A-CH)) sind kommunikative Kompetenzen zunehmend fest in Lehre und Prüfungen verankert. Zur Unterstützung der weiteren curricularen Entwicklung bezüglich kommunikativer Kompetenzen arbeitet die Umfrage des GMA Ausschusses „Kommunikative und soziale Kompetenzen“ (KusK) systematisch auf, in welchem Umfang und in welcher Form unterrichtet und geprüft wird.

**Methodik: **Der iterativ in Zusammenarbeit mit „KusK“ entwickelte Onlinefragebogen umfasst 70 Fragen zu Unterricht (n=14), Prüfungen (n=48), lokalen Bedingungen (n=5) und drei Felder für sonstige Anmerkungen. Pro Standort wurden zwei bis drei Personen, die mit dem Curriculum vor Ort vertraut sind, zur Teilnahme an der Umfrage eingeladen.

**Ergebnisse: **Es beteiligten sich 39 medizinische Fakultäten (40 Studiengänge) an der Umfrage. In allen Studiengängen werden kommunikative Kompetenzen unterrichtet. Zehn Studiengänge haben ein longitudinales Curriculum für kommunikative Kompetenzen, in 25 Studiengängen existiert dies teilweise. 16 der 40 Studiengänge orientieren sich am Baseler Consensus Statement. In über 80 % der Studiengänge werden kommunikative Kompetenzen im zweiten und dritten Studienjahr unterrichtet. Fast alle arbeiten mit Simulationspatienten (n=38) und Feedback (n=37). Geprüft wird nur summativ (n=11), nur formativ (n=3) und sowohl summativ als auch formativ (n=16). Am häufigsten wird im vierten bzw. fünften Studienjahr geprüft (n=22 bzw. n=20). Neben schriftlichen Tests (n=15) und Referaten (n=9) sind vor allem praktische Prüfungen implementiert (OSCE (n=31); WPA (n=8)), meist mit selbst entwickelten Beurteilungsskalen (OSCE: n=19). Bezüglich der Schulungen der Prüfer sowie der Art und Weise der Ergebnisrückmeldung an Studierende besteht eine hohe Varianz.

**Schlussfolgerung: **Der Unterricht von kommunikativen Kompetenzen wurde an allen beteiligten 39 medizinischen Fakultäten implementiert. Kommunikative Kompetenzen werden in medizinischen Curricula in D-A-CH überwiegend in kleinen Gruppen unterrichtet und mittels OSCE geprüft. Herausforderungen für die weitere curriculare Entwicklung liegen im Ausbau von Feedback, der kritischen Überprüfung von geeigneten Assessmentstrategien und in der Qualitätssicherung der Prüfungen.

## Introduction

Communicative competencies have been taught and integrated into pass-relevant exams in many countries for years now [1], [2], [3], [4], [5]. In German-speaking Europe, curriculum development has been supported and facilitated in recent years by the defining of learning objectives catalogues, such as the Basler Consensus Statement,[6], [7] or by the development of a longitudinal, Bologna-compatible model curriculum [8]. A survey by Roch et al. [9] showed that the extent of instruction in doctor-patient communication varied between the different medical schools. The question of the degree to which this instruction was oriented on a learning-objectives catalogue and on which models and theories it was based remained open. The issue of assessment was only briefly outlined in the von Roch survey, making differentiated assertions regarding assessment methods infeasible.

In the training context, it is becoming increasingly important to develop appropriate assessment strategies for assessing not only the efficacy of communication training but above all the learning progress of students [10]. Furthermore, communicative competency evaluation is considered to be a long-term strategy for the implementation of communication training in the framework of regular instruction [11]. The joint planning of courses as well as the corresponding examinations were highlighted by Bachmann et al. [8] in 2009.

The call for instruction and assessment of communicative competencies in medical education was augmented by the stipulations of the 2012 revision of the German Medical Licensure Regulation, requiring the testing of communications skills within the state exams (retrieved September 11, 2014, from http://www.gesetze-im-internet.de/bundesrecht/_appro_2002/gesamt.pdf). Since the introduction of the “clinical skills” test within the scope of the Swiss examination procedure, communicative competencies have also become relevant to passing Switzerland’s state exams [12]. In Austria, communicative competencies are incorporated in the “Austrian Competence Level Catalogue for Medical Skills” (retrieved December 14, 2014, from http://kpj.meduniwien.ac.at/fileadmin/kpj/oesterreichischer-kompetenzlevelkatalog-fuer-aerztliche-fertigkeiten.pdf). To date, however, only scarce data are available regarding the assessment of communicative and social competencies in German speaking faculties in these three countries.

According to Weinert [13], communicative competencies can be seen as skills and conduct that contribute to problem solving – normally patient problems. Hence, all established assessment strategies may be applied in order to evaluate communicative competencies or segments of these competencies: written and computer-based tests (e.g. multiple choice questions - MCQ) [14], practical exams in simulated situations (e.g., objective structured clinical examinations - OSCE) [15], [16] and evaluations in real situations, i.e., workplace-based assessment (WBA) [17]. Papers, presentations, case summaries or online or paper-based portfolios (retrieved September 3, 2014, http://methodenpool.uni-koeln.de/portfolio/frameset_portfolio.html), [18], [19] are also principally conceivable as evaluation formats.

A recent survey by Laidlaw et al. [20] showed that the average frequency of clinical communication assessment came to ten test intervals per degree programme. Students were tested an average of 2.4 times per year. The main assessment point was in the final academic year. On the average, 3.1 examination methods were used per course, while only 26% of the courses were tested using one single method. OSCEs were implemented most frequently. Written exams (MCQ, short-answer questions, portfolios) were particularly prevalent in the first years of training. OSCEs and WBA were used at greater frequency in later years of study [20]. At present, there are no data available for German-speaking Europe from which a similar development might be deduced.

The objective of the present study is, in cooperation with the committee for “Communicative and Social Competencies” (*KusK*) of the German Society for Medical Education, to survey the current practice of the teaching and assessment of communicative competencies in medical studies programmes in German-speaking Europe.

The study addresses the following questions:

1. How are communicative competencies taught in programmes of medical study in German-speaking Europe?

- When does instruction take place?
- Which teaching formats/methods are being implemented?
- What is being taught?
- How is feedback applied in instruction?

2. How are communicative competencies assessed in programmes of medical study in German-speaking Europe?

- When does assessment take place?
- Which assessment formats / methods are being implemented?
- How is assessment quality assured?
- How is feedback applied in assessment?

## Methods

An online survey was conducted in every medical faculty, medical college or medical university (n=43) in Germany, Austria and German-speaking Switzerland offering a complete curriculum at the time of survey. The cross-sectional study aimed at an exhaustive inventory. 

The questionnaire for the survey was developed in a multistage process in consultation with the *KusK* committee. An initial version of the questionnaire was created in January 2013 using the open-source software “Lime Survey” (version 1.92+). The questionnaire was sent by e-mail to the 41 participants of the February 2013 *KusK* committee workshop in Vienna. Twenty-two participants from Germany, Austria and Switzerland responded, providing answers from 18 different faculties in total. The results were presented in the course of the workshop and discussed and modified in the “Assessment” workgroup. 

After further revision, the final questionnaire contained 70 items (see Attachment 1). Twelve of the questions concerned the teaching of and 43 the assessment of communicative competencies (see Table 1 (Tab. 1)).

For each site (medical faculty, medical university, medical college), two to three contact persons were sought to fill out the questionnaire for their respective faculty. An essential criterion in the search was the respective contact person’s thorough familiarity with the courses and exams/assessments in which communicative competencies play a role. Many of the reference persons could be directly contacted by active committee members. In August 2013, contacts from 43 German-speaking sites were approached by e-mail and asked to process the survey. All of the participants who had taken part in the pilot survey were able to access the answers they had provided at the time of its implementation. In cases in which two separate responses were received from a single site, the contact persons were approached again and asked to determine a joint final version. Once the survey was concluded, the results were exported to Microsoft Excel 2010 and descriptively evaluated. All information was treated confidentially, and the participants were assured that the results would be reported in anonymous form. 

### Ethics commission approval

A request for approval of the survey was placed with the ethics commission of the medical faculty of the Ludwig Maximilian University of Munich. The ethics commission issued a declaration of no-objection (UE No. 140-13).

## Results

### Response return and data on site and degree programme

Thirty-nine of the 43 sites approached (medical faculties/universities/colleges) participated in the survey. This number represents a 91% return (see Table 2 (Tab. 2)). At one site, the survey was processed by two different programmes in human medicine (one regular course of studies and on model programme), resulting in information for 40 programmes from only 39 sites.

**Figure 1:**
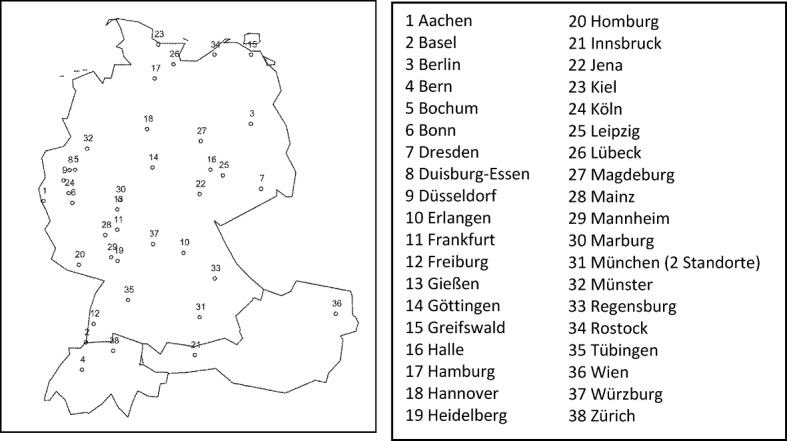
Overview of survey-participating cities

An overview of the cities participating in the survey is offered in Figure 1 (Fig. 1). In Munich, both the Ludwig Maximilian University and the Technical University participated in the survey. 

Between 42 and 900 students are taught in the 40 degree programmes from which the data herein were retrieved. This comes to an average (median) of 265 students per academic year.

#### Contact person’s self-estimation of their knowledge regarding the instruction and assessment of communicative competencies in their own degree programmes 

Of the 40 contact persons, more than half (55%) estimated their familiarity the communicative-competency related aspects of the entire respective curriculum to be good, 30% confirmed good familiarity with the majority of the relevant courses, while 15% reported familiarity with only part of the relevant courses. 

Of the 32 contact persons whose curricula included pass-relevant communicative competencies assessments, 47% estimated their knowledge levels of the exams in the area of communicative competencies to be good. Twenty-eight percent reported a good overview of the exams, while 25% reported a general overview of the relevant tests in this area. 

#### Transmitting communicative competencies

##### When does instruction take place?

At the time of the survey, communicative competencies were being taught in all of the participating degree programmes. Ten of the 40 degree programmes indicated that all of their courses in communicative competencies were coordinated in accordance with a longitudinal curriculum. A longitudinal curriculum existed in part or had just been implemented in 25 degree programmes, while five programmes reported having no longitudinal curriculum (see Table 3 (Tab. 3)). 

Over 80% of the programmes emphasised instruction during the second and third years. Communicative competencies were taught in the sixth year in 38% of the programmes. Degree programmes with fully or partially implemented longitudinal curricula indicated comparable distribution of instruction. Instruction in the first, second and sixth year of studies was comparatively rare in programmes without a longitudinal curriculum (see Table 3 (Tab. 3)). A detailed breakdown of the distribution of instruction by academic year is offered in Attachment 2.

##### Which teaching formats/methods are being implemented?

**Teaching formats**

In 18 programmes, instruction took place in three different teaching formats: lecture and seminar format (max. 20 participants), small-group format (max. 6 participants). In 12 programmes, two different teaching formats were used: seminar format (n=12), small-group format (n=10), lecture format (n=2). Ten programmes used only one teaching format – usually seminars (n=8). 

Overall, the most frequently (38 of 40 degree programmes) implemented form of instruction of communicative competencies was the seminar format (between six and 20 participants). Thirty programmes indicated that instruction took place in groups with less than six students (e.g., practical exercises). Multiple entries were possible.

**Teaching methods**

Role-play with simulated patients (SP, n=38) and feedback (n=37) were the most frequently deployed forms of instruction. A detailed breakdown of the implemented teaching methods is available in Table 4 (Tab. 4). Furthermore, discussions with representatives of self-help groups and video feedback were indicated in the answers to open-ended questions.

##### What is being taught?

In 28 of the 40 degree programmes (70%), teaching staff referred to a superordinate model, a learning-objectives catalogue or other instruments of curriculum planning for orientation in instructional content design. Cited 16 times, the Basler Consensus Statement7 was by far the most frequently indicated learning objectives catalogue. The German National Competency-Based Learning Objectives Catalogue NKLM [21] (retrieved September 14, 2014, from http://www.nklm.de) was named five times. Further learning objectives catalogues were: self-developed learning objectives catalogues, the content outline of the German Institute for Medical and Pharmaceutical Examination Questions (IMPP; retrieved November 14, 2014, from https://www.impp.de/internet/de/medizin/articles/gegenstandskataloge.html) and the CanMEDS Physician Competency Framework of the Royal College of Physicians and Surgeons of Canada [22].

Within the framework of instruction, various techniques or schemes were implemented (see Table 5 (Tab. 5)). The SPIKES model [23] for delivering bad news and the NURSE model [23], [24] for dealing with emotions were cited most frequently. 

##### How is feedback applied in instruction?

Thirty-seven of the 40 programmes reported that feedback was used in the teaching and assessment of communicative competencies. All of the 37 programmes used feed back in their instruction (classroom teaching). In 18 of the programmes, feedback was also used in assessment, and five of the 37 employed it in an e-learning framework.

Teaching staff provided the feedback in all 37 programmes in which it was implemented. Additional feedback came from fellow students in 35 programmes and from SPs in 31 programmes. Real patients were rarely (n=4) named as sources of feedback. None of the programmes indicated nursing staff as sources of feedback. The situations on which feedback was given are detailed in Table 6 (Tab. 6).

Twenty-six of the 37 programmes (70%) using feedback employed observation forms in the structuring of feedback. Self-developed forms were predominantly used (n=25). In six programmes, the Calgary Cambridge Guide [25], [26] was used. Multiple entries were possible. 

The theoretical model on which the delivery of feedback was based in the 37 programmes in which feedback was implemented was also surveyed. Responses to this query were received from 19 programmes. Multiple entries were possible. Thirteen programmes made no response to the query. Four programmes responded that they did not know, and one programme replied that there was no model/theory base. 

The most frequently named models or theories were theme-centred interaction [27] (n=9), the sandwich model [28], [29] (n=4) and Schulz von Thun [30] (n=3). All other theories or models were indicated once or twice. Furthermore, a “combination” or “a sound mixture” of various models and theories was reported for five of the degree programmes.

#### Assessing communicative competencies

Communicative competencies were tested in 88% (n=35) of the 40 degree programmes. Communicative competencies were evaluated summatively (relevant to passing) in 32 of these programmes, while in 14 programmes evaluation was formative (multiple entries possible). Among these, 16 programmes employed both evaluation modalities and 14 programmes only one (exclusively summative: n=11; exclusively formative: n=3). Five programmes reported summative evaluations but could not provide an answer concerning the implementation of formative evaluations (formative: “no knowledge”, n=5).

##### When does assessment take place?

Summative and formative evaluations were conducted most frequently in the fourth or fifth academic year. The distribution of evaluations throughout the years of study is detailed in Appendix 3. In 21 programmes, marks were given in evaluations in which communicative competencies were covered along with other competencies. Six programmes reported giving no marks, while four reported giving a single mark solely for communicative competencies. 

In 29 of the 40 degree programmes, there was a central examination department or faculty deanery that assists teaching staff in the preparing, implementation and/or evaluation of examinations. A negative response was received for nine programmes. The question could not be answered for two of the programmes.

##### Which assessment formats are being implemented?

Of the various assessment formats, OSCEs were named most frequently (31 entries). Portfolios were rarely used (see Table 7 (Tab. 7)). 

##### Paper-based and computer-based assessments

For ten of the twelve programmes reporting summative evaluations, it was indicated that the paper-based and computer-based evaluation of communicative competencies that were relevant to passing were administered in combination tests (i.e., together with other formats or competencies). Pure communication tests were indicated for six programmes. Predominantly multiple choice questions were used, testing only factual knowledge. Table 8 (Tab. 8) details question types/stimuli and answer formats as well as the number of entries.

The examiners responsible for preparing and conducting the tests in nine programmes came from the field of medical psychology and sociology (n=9), followed by psychosomatics (n=6), general medicine (n=4), internal medicine as well as surgery and psychiatry (n=3 for each), paediatrics (n=2), orthopaedics, ENT, ophthalmology, neurology, medical ethics, palliative medicine, medical students and pedagogically trained staff from other professions (n=1 for each). In six programmes, the examiners also were also conducted instruction; in nine programmes they only instructed to some extent. For one programme, it was reported that examiners did not teach at all. “Varies greatly” or “unknown” was indicated twice. 

##### Objective Structured Clinical Examination (OSCE)

Of the 31 programmes in which OSCEs [15], [16] were conducted, the number of OSCEs varied between one and twelve. In 68% of the programmes, communicative competencies were tested in one or two OSCEs (see Table 9 (Tab. 9)). 

The OSCEs in which communicative competencies were tested were conducted primarily in one or two different years of study (n=22). In one programme, OSCEs were held in five different years of study. 

The individual OSCE stations predominantly (n=26) comprised integrated stations (various skills - e.g., case history, examination and communication - are tested in one station). Furthermore, communicative competencies were tested in stations in which various competencies were being tested simultaneously (e.g., physical examination, case history, resuscitation; n=20). OSCE stations exclusively testing communicative competencies were less frequent (n=7).

Student performance was assessed most frequently by means of unvalidated instruments (n=19). In 18 programmes, instruments were used that had been theoretically tested beforehand. These were either self-developed instruments (n=10) or published instruments (n=8). For five programmes, the question regarding evaluation instruments could not be answered. Multiple entries were possible. 

Six of the instruments indicated were validated assessment instruments, the Berlin Global Rating [31] (n=4) and the Calgary Cambridge Guide [25], [26] (n=2) being the most frequently named. The Frankfurt Observer Communication Checklist (FrOCK) [32], the communication evaluation form from the University of Cologne (KEK) [33], the Swiss performance-based rating (Eidgenössisches Verhaltensbasiertes Rating) [34] and the Vienna performance-based rating (global rating scales ÄGF –A) [35] were each named once. 

Most of the programmes kept detailed checklists independent of the applied instrument (n=17). Global assessments (n=10) and a combination of both (n=13) were used less frequently. In addition, one programme employed a single-point checklist, and another programme used a personal progress reflection.

The examiners were predominantly physicians (n=29), followed by professionals from social sciences such as psychology, educational science, and sociology (n=15). In five programmes, SP were installed as examiners, and four programmes used students. 

The configuration of physicians was usually a mix of representatives of different specialist fields (n=15). General medicine (n=10) and internal medicine (n=7) were the most frequently named. All other specialist fields were named five times or less: surgery, psychosomatics, medical psychology and medical sociology (in Germany), paediatrics, gynaecology, psychiatry, anaesthesiology, palliative medicine, hygiene, ENT, neurology, ophthalmology. 

In eight programmes, instruction and assessment were conducted by the same staff, while 20 programmes reported this to be only partially the case. One programme reported that examiners and teachers were not one and the same, while two programmes were not able to provide a response. 

##### Workplace-Based Assessment (WBA)

WBA [17] was implemented in eight degree programmes, using both unvalidated (n=4) and test-theory controlled instruments (self-developed and non-self-developed; n=2 for each). Non-self-developed instruments used were the Mini-CEX (Mini Clinical Examination; n=3), the DOPS (Direct Observation of Procedural Skills; n=1) and a modified encounter card (n=1) [17]. WBA was not administered as a pass-relevant individual assessment in any of the programmes. In three programmes, the WBA together with other assessments was relevant to passing. 

##### Portfolio 

The portfolio (retrieved Sep 9 2014, from http://methodenpool.unikoeln.de/portfolio/frameset_portfolio.html), [18], [19] was used as an assessment format for communicative competencies in two programmes. Clinical skills both including and excluding communicative competencies were evaluated by portfolio. In one of the programmes, the portfolio comprised six to ten individual certificates, according to the stipulations of the various practical stations. The other programme could not provide any details in this regard. Physicians fulfilled the function of examiner in one programme; the other programme could not provide these details. The portfolio was neither relevant to passing nor part of a pass-relevant evaluation in either programme. 

#### How is assessment quality assured?

In the 32 programmes with communicative competencies assessments relevant to passing, norm-referenced methods [36] were predominantly used to establish a cut-off score (e.g., a specified percentage of 60%). Criterion-referenced methods [36] were only used with OSCEs. The methods used for the paper-based and computer-based evaluations and OSCEs are detailed in table 10 (Tab. 10). No methods were reported for WBA. 

For the quality assurance of the most frequently used assessment formats (paper-based and computer-based assessments and OSCEs), various methods were used (see Table 11 (Tab. 11)). 

Examiner training was the most frequently employed measure for quality assurance, particularly with the OSCE. The participation conditions for examiners varied between absolutely voluntary participation to obligatory participation with corresponding certification. Training duration varied between 30 minutes and two days. For the most part, training of one to four hours was offered (n=11). 

The following formats for examiner training were reported: classroom lectures, small group work, training with simulated patients, video training, peer observation and individual coaching. Technical training, instrument testing, observation with feedback and station-specific briefings took place during the training. Additionally, “refresher courses” for experienced examiners were conducted in two of the programmes. The proximity in time to the administering of the assessments was predominantly reported as being one week before to directly before the assessment. Cases in which examiners with several years of experience required no further training were also reported (n=2). 

#### How is feedback applied in assessments? 

##### Feedback in the context of paper-based and computer-based assessments 

In two of the programmes, all of the students received detailed feedback following the assessment. Detailed feedback for subgroups (e.g., students who did not pass) was made available in four of the programmes. Students in all of the programmes in which paper-based and computer-based assessments were implemented received feedback in the form of overall results. In four programmes, the feedback was delivered in the form of results for the individual questions. Multiple entries were possible.

##### Feedback in the context of OSCEs

Feedback on OSCE results was given in the form of overall results to students in 24 of the 31 programmes in which this assessment format was implemented. In 17 programmes, feedback was also given in the form of results for each individual station. Detailed feedback was given to all of the students in eight programmes, and twelve programmes gave detailed feedback to certain subgroups (e.g., students who had not passed). Additionally, oral feedback subsequent to the individual stations, electronic feedback and feedback in terms of individual coaching was given. Multiple entries were possible. 

## Discussion

The objective of the survey was a complete inventory of the transmission and assessment of communicative competencies in German-speaking Europe. This is the first survey that has systematically covered both of these subdomains in Germany, Austria and German-speaking Switzerland. With response returns of over 90%, sound informative value of the results for German-speaking Europe can be assumed. The high rate of participation in the survey can be attributed to the now broad acceptance of the subject and the good networking of instructors through the *KusK* committee of the *GMA*. 

The most significant result of the survey is the finding that in 2013 all of the sites participating in the survey, irrespective of the size of the programme, implemented communicative competencies instruction in medical studies. This was not the case at all sites in 2010 [9]. Furthermore, communicative competencies are assessed in almost 90% of the programmes. In notable contrast to the study by Laidlaw [20] however, communicative competencies are instructed and assessed less frequently in German-speaking countries than in the United Kingdom. According to Laidlaw et al. [20], students were assessed 2.4 times per academic year on average in the United Kingdom. In German-speaking Europe, only three programmes assessed communicative competencies in four or five academic years at the time of the survey. In 22 programmes, assessment took place in two or three academic years. Furthermore, the main assessment point of communicative competencies in the United Kingdom was in the final year of studies. In German-speaking Europe, the main assessment took place in the fourth or fifth academic year.

Longitudinal, cross-semester curricula were already in place at many of the survey sites. At the majority of sites where they were not yet in place, they were either in the planning or in the implementation phase. The most frequently employed learning-objectives catalogue was the Basler Consensus Statement, a position paper from the German Society for Medical Education *(GMA)* from the year 2008 [6]. This also speaks for the success of the now long-standing committee work of the *GMA*.

The use of SPs in communications instruction has largely prevailed in German-speaking Europe, as has the use of feedback. Instruction in groups with a maximum of 20 students was the most frequent form of instruction, followed closely by small-group instruction with groups of six students or less. Particularly in light of the resources and personnel necessary, this is a development that was not foreseeable several years ago. 

The most frequently used communications model or flowchart was the SPIKES protocol [23]. For other consultation occasions - obtaining information (medical history), informing the patient or regular consultation, for example – no model seems to have prevailed in the same manner. Less situation-specific communication models, such as the WWSZ model or the NURSE model [24], were also implemented, however less frequently than the previously indicated SPIKES protocol. This could be explained by the fact that breaking bad news represents an obvious difficulty entailed in the medical practice of instructors and students that is easily comprehensible – in contrast with general consultations or dealing with emotions, which may be seen as less specific and hence less urgent. 

The preconception that communication is imparted exclusively through the disciplines of psychology, psychiatry or psychosomatics, for example, is refuted by the broad discipline range of the instructors. Thus, the recommendation to execute the communication of information through various disciplines is being followed at many sites in German-speaking Europe in the meantime [11].

In 2013, 80% of the sites assessed communicative competencies predominantly as relevant to passing and most frequently by means of OSCEs. Paper-based and computer-based assessments were usually implemented in the first two years of study, while OSCEs were used mostly in the fourth and fifth years. 

For the most part, communicative competencies were assessed together with other clinical competencies, pure communication OSCEs being rather the exception. This adheres to current recommendations that communicative competencies be integrated and not separated in instruction and assessment [11], (retrieved May 4, 2015, from http://www.each.eu/teaching/can-teach-offer/assess/assess-general-principles-assessment/). In the evaluation of student performance, checklists as well as global assessments and hybrid forms were used. No clear trend in this area has been established yet. 

For the most part, multiple choice questions relating to factual knowledge were used in the written assessments (paper-based or computer-based). On the one hand, it can be seen as a success that the subject of communicative competencies has made its way into what is doubtless a very prevalent and established assessment format in the faculty, on the other, however, the adequacy of a focus on factual knowledge for establishing clinical knowledge and allowing the transfer of communicative competencies to daily clinical life must be critically scrutinised. An enhancement with context-rich question types would be a desirable change in this respect. 

The establishment of a cut-off score by means of criterion-oriented methods was practiced at only a few of the sites. International recommendations have apparently yet to reach German-speaking faculties to an adequate extent [36], [37]. This may be due in part to the respective examination regulations of the medical schools in which the cut-off score can be limited to norm-referenced methods (e.g., pre-determined points or percentages, regardless of the examination in question – for instance, 60%). The introduction of review procedures, writing workshops, statistical analyses or examiner training represents another aspect of assessment quality assurance. The implementation of these varied strongly, especially with respect to examiner training – the diversity is not otherwise explainable. The need for further research was particularly evident concerning OSCEs. 

More recent assessment formats, seemingly more appropriate for formative assessments, such as the WBA or the portfolio, showed only scattered implementation. This may be due to the fact that formative assessment is generally less frequent in German-speaking countries. Considering, however, the use of feedback as an effective teaching and learning format already being implemented in many places, it is exactly these assessment formats that should be more widespread. Additionally, in light of the current change from “assessment of learning” to “assessment for learning” [38], the progression towards the promotion of enduring and reflective learning would be very beneficial for precisely these formative formats as accompanying instruments. 

## Limitations

Despite the relative lengthiness of the 70-item questionnaire, not all of the questions necessary for a complete picture of the situation could be asked. In preparing the questionnaire, priority was given to information needs as well as the practical manageability of the instrument. The considerable surplus of items in the area of evaluations/assessments compared to those used to acquire date in the area of instruction is due to the fact that only scarce systematic information was available on the assessment of communicative competencies in German-speaking countries. The consequence of this was that relatively few questions on the transmission of communicative competencies could be asked (see Attachment 3). 

It is possible that the subject of communication was also taught and assessed in other courses that were not captured within the scope of the survey. For example, it is conceivable that bedside teaching was used for this subject. Thus, it must be taken into account that not all courses in which communication was incorporated were reported, but that the emphasis was moreover on courses that dealt primarily with the subject of communicative competencies. Furthermore, the contact persons at the different sites respectively reported varied levels of knowledge regarding the instruction and assessment of communicative competencies in their programmes. Consequently, the survey cannot claim to offer a complete picture of the curricula for communicative competencies at all of the participating sites. Particularly with regard to subtopics such as team communication or interprofessional communication that have gained in significance in recent years, information may not have been captured by the survey. These emerging topics in clinical communication should be subjected to a systematic analysis in the years ahead.

Furthermore, the survey could not capture the quantitative student-teacher-ratio, nor was it possible to gather information on the number of communicative-competency instruction hours that the individual students had in the framework of compulsory training during their studies. The subject of “resources” for instruction and assessment was also only rudimentarily probed. An analysis of which influential factors for the implementation of a longitudinal communication curriculum are identifiable would be of further interest. These could aid faculties still in the implementation phase in advancing the successful development of a curriculum. 

## Perspectives

The survey was limited to curricula for human medicine in German-speaking Europe. Further surveys could be used to acquire information regarding the instruction and assessment of communicative competencies in other health professions. The questionnaire has already been adapted and conducted for dental medicine [39]. 

An important insight from the survey was that there is apparently no consistent set of recommendations in German-speaking Europe for the use of feedback. From the heterogeneity of the answers, the conclusion can be drawn that, in contrast with the SPIKES protocol, no feedback model has prevailed that has allowed for a standardisation of instruction. A KusK task force is currently addressing this subject and formulating relevant recommendations on the basis of the latest literature and practical examples for training. 

The answers to the questions regarding assessment quality assurance were also notably varied. This applies particularly to examiner training, above all for the most frequently employed assessment format, OSCE. The formulation of recommendations on the basis of evidence would be a desirable development. 

On the whole, a widely varied pallet of instruction and assessment in German-speaking countries was evidenced. This can be interpreted as a great potential in the scheme of tertiary education, demonstrating a capacity for development and for the analysis and comparison of various approaches, with all of their respective advantages and disadvantages. Additionally, this shows room for new concepts and ideas. It can, however, also be interpreted as a possible danger, demonstrating a potential obstacle to the mobility of students between the variant medical curricula in German-speaking Europe. 

For these issues, the present study offers a basis upon which further enquiry can be constructed as well as a basis of orientation for curriculum design. 

## Acknowledgements

Our particular thanks goes to all those who participated in the creation of the survey and to those who provided their responses. We would also like to offer special thanks to the members of the German Society for Medical Education’s (GMA) committee for communicative and social competencies as well as the participants from the “assessment” task force at the February 2013 workshop in Vienna. We thank Johanna Feckl for her proofreading and her support in formatting. Further thanks go to Matthias Holzer for his help in creating the map. For his support in this work, our sincere thanks go to Martin Fischer as well. 

## Competing interests

The authors declare that they have no competing interests. 

## Supplementary Material

The following is the survey questionnaire for communicative competencies in medical studies in German-speaking Europe.

Data on the years of study in which communicative competencies are instructed in the curricula and whether a longitudinal communication curriculum has been implemented in full, in part or not at all (academic year=AJ)

Evaluations timeframe for summative (pass-relevant; “s”) assessments and formative (not pass-relevant; ”f”) assessments over the years of study

## Figures and Tables

**Table 1:**
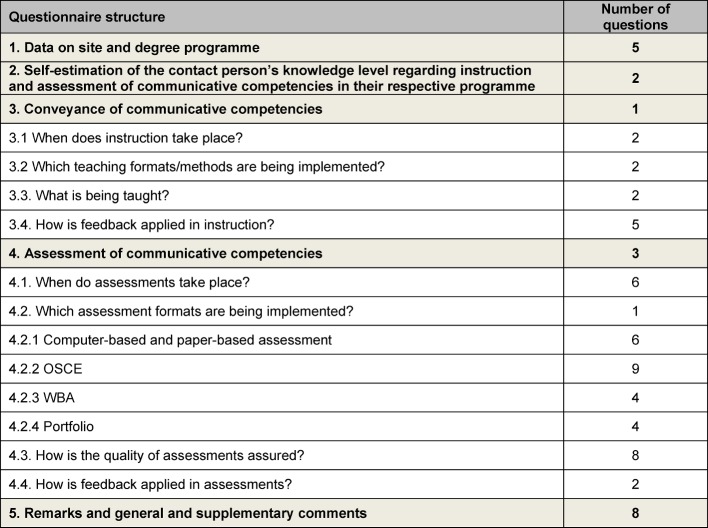
Distribution of the 70 survey questions according to the issues addressed in the present study

**Table 2:**
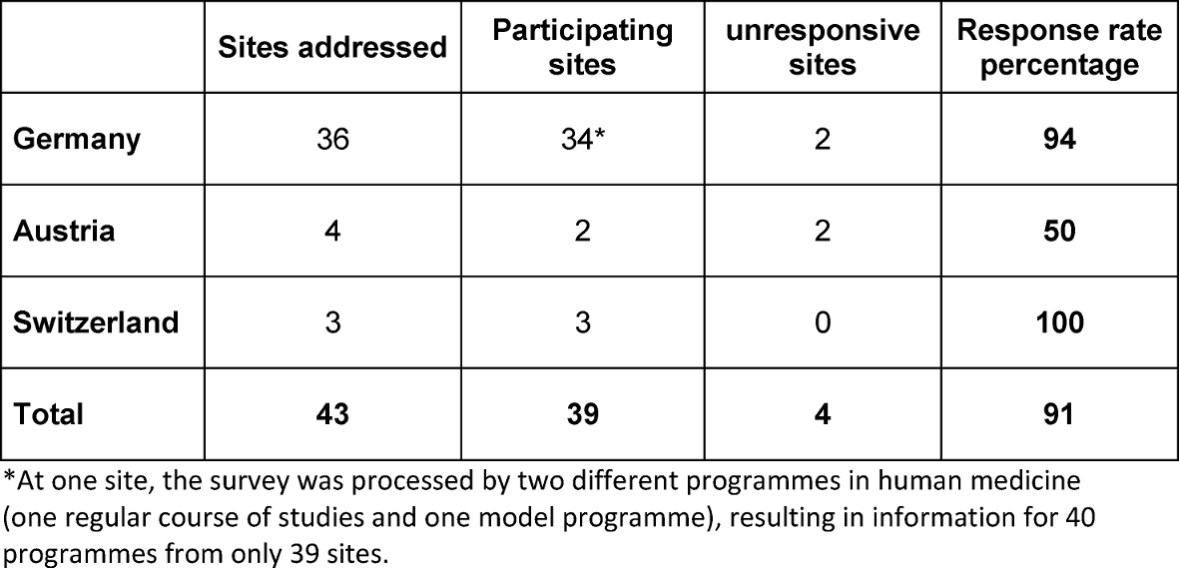
Online-survey participation by site (medical faculty/university/college) in German-speaking Europe

**Table 3:**
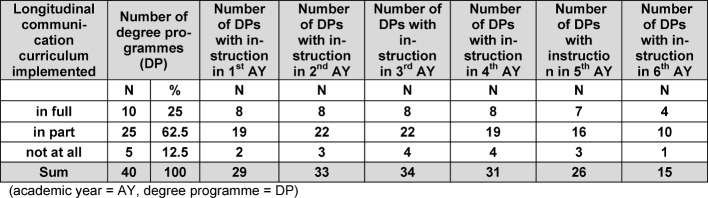
Data on curricular instruction of communicative competencies by academic year and the full, partial or non-implementation of a longitudinal communication curriculum (academic year=AY, degree programme=DP).

**Table 4:**
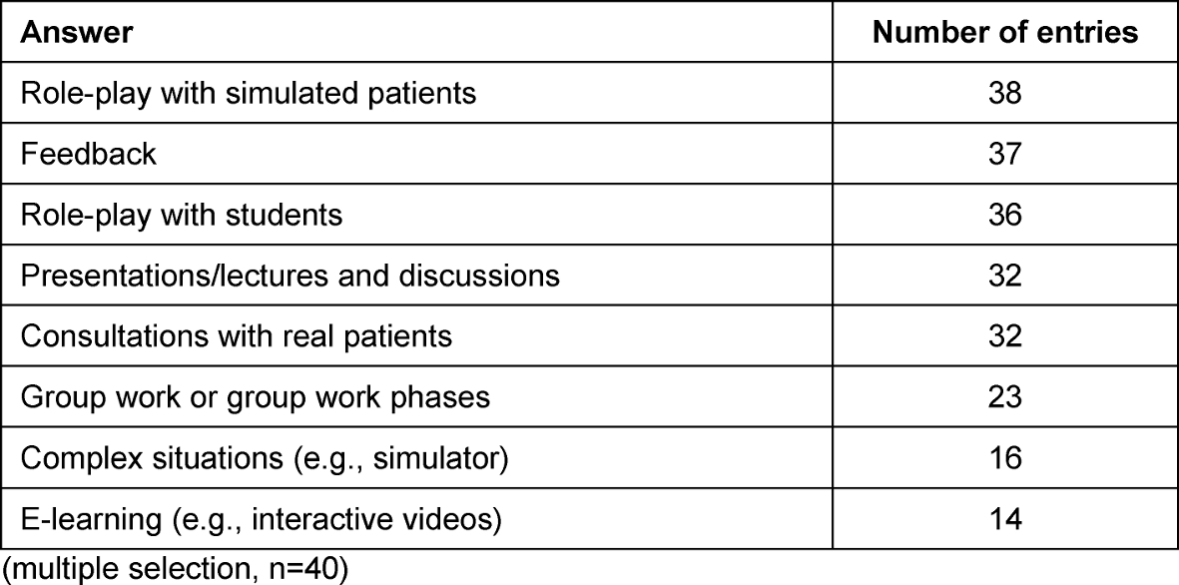
Number of answers to the question: “Which pedagogical elements are used?” (multiple selection, 40 participants)

**Table 5:**
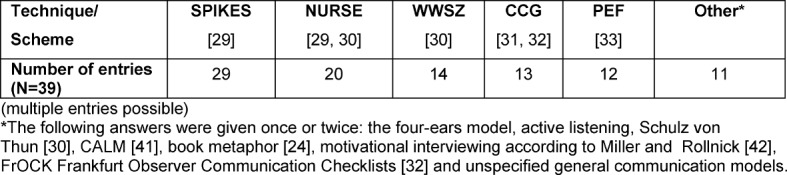
Answers to the question: “Are specific techniques, schemes or the like imparted?” (multiple entries possible)

**Table 6:**
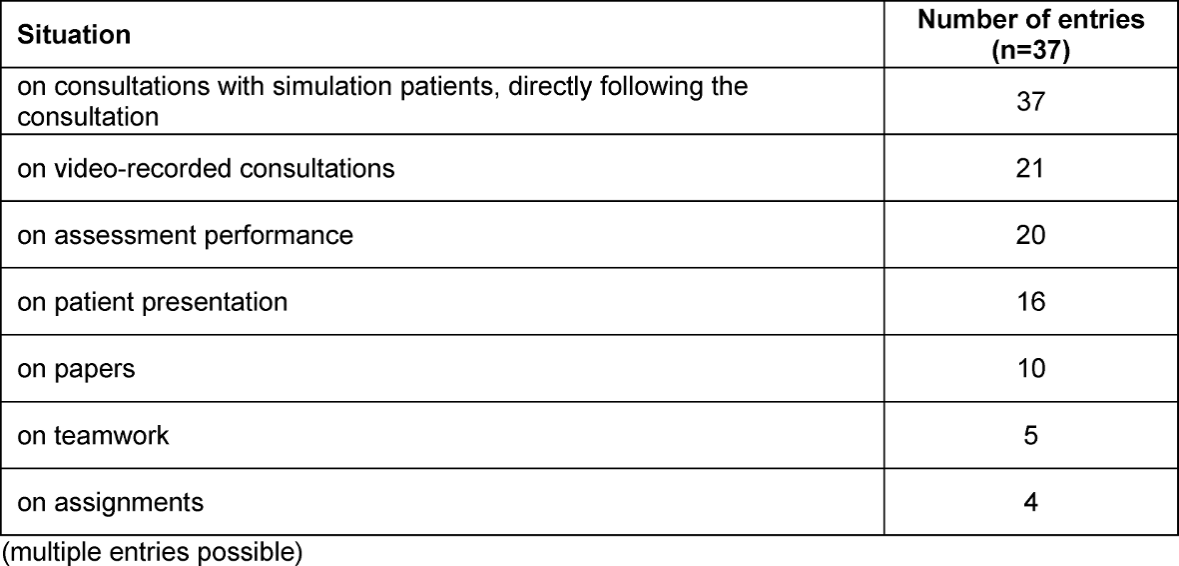
Number of answers to the question: “What do students receive feedback on?” (multiple entries possible)

**Table 7:**
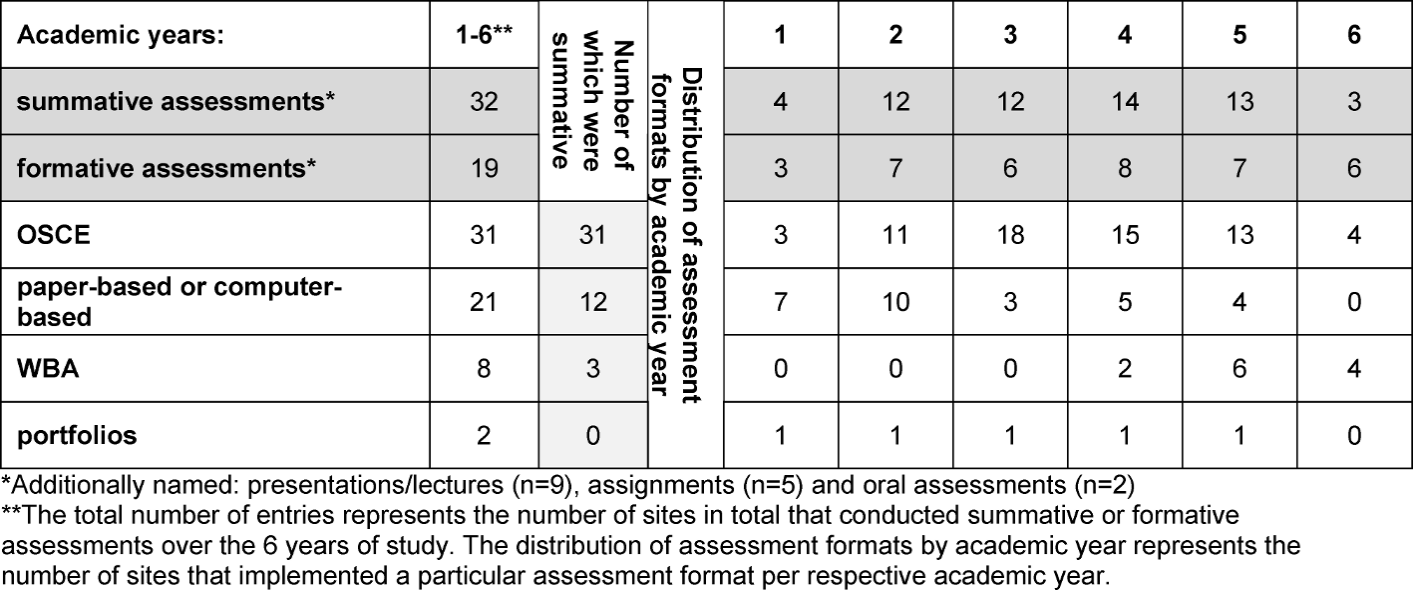
Distribution of individual assessment formats over the course of the years of study (n=35). Multiple entries were possible. The number of entries per academic year is detailed here.

**Table 8:**
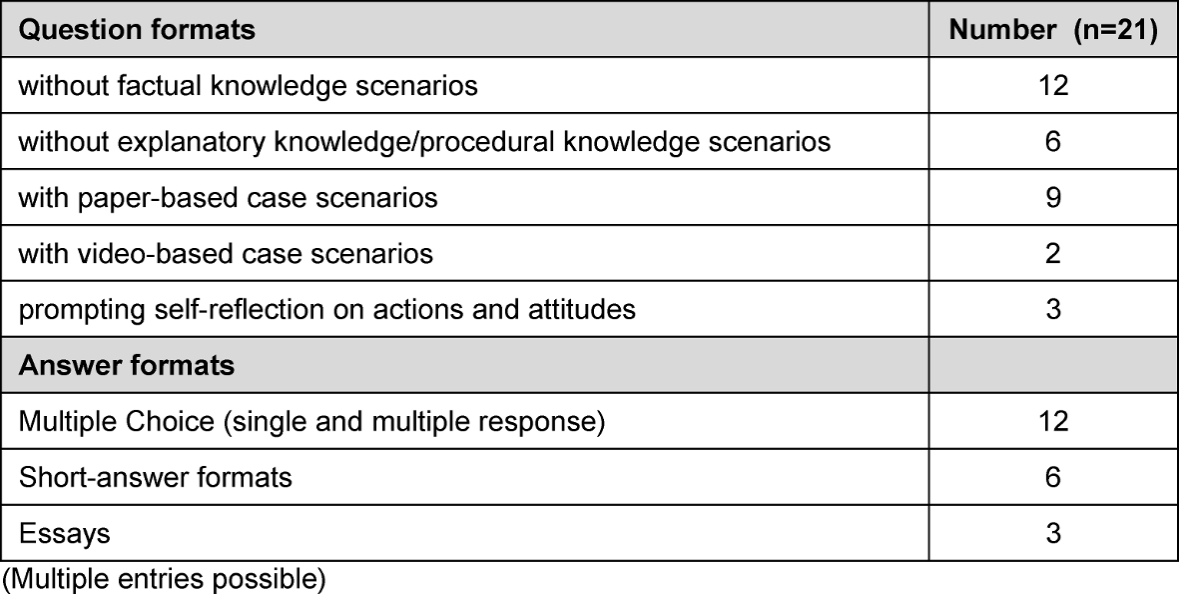
Question types/stimuli and answer formats used in paper-based and computer-based written tests. (Multiple entries possible)

**Table 9:**
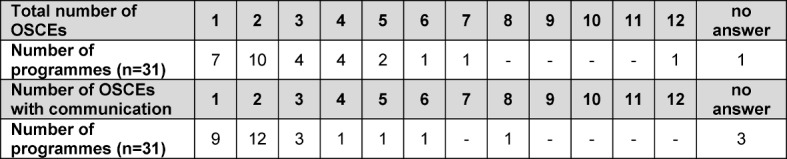
Total number of OSCEs and number of OSCEs in which communicative competencies were tested per degree programme

**Table 10:**
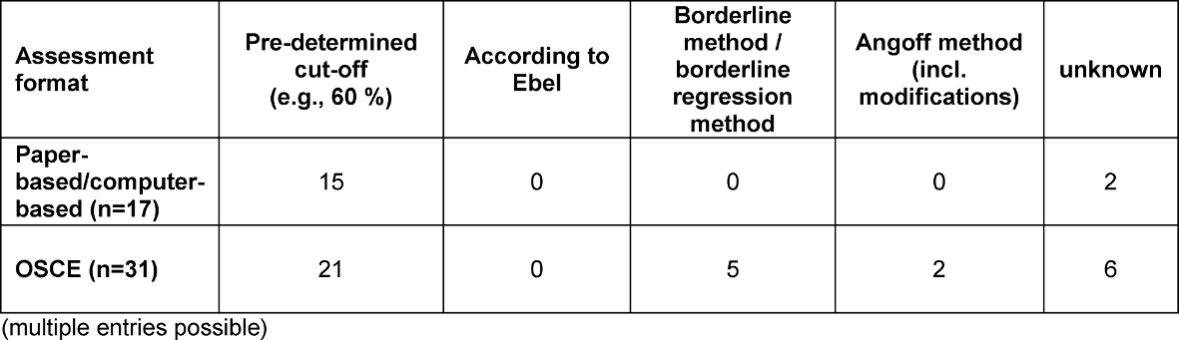
Methods employed for establishing a cut-off score [39] for the summative assessments (multiple entries possible)

**Table 11:**
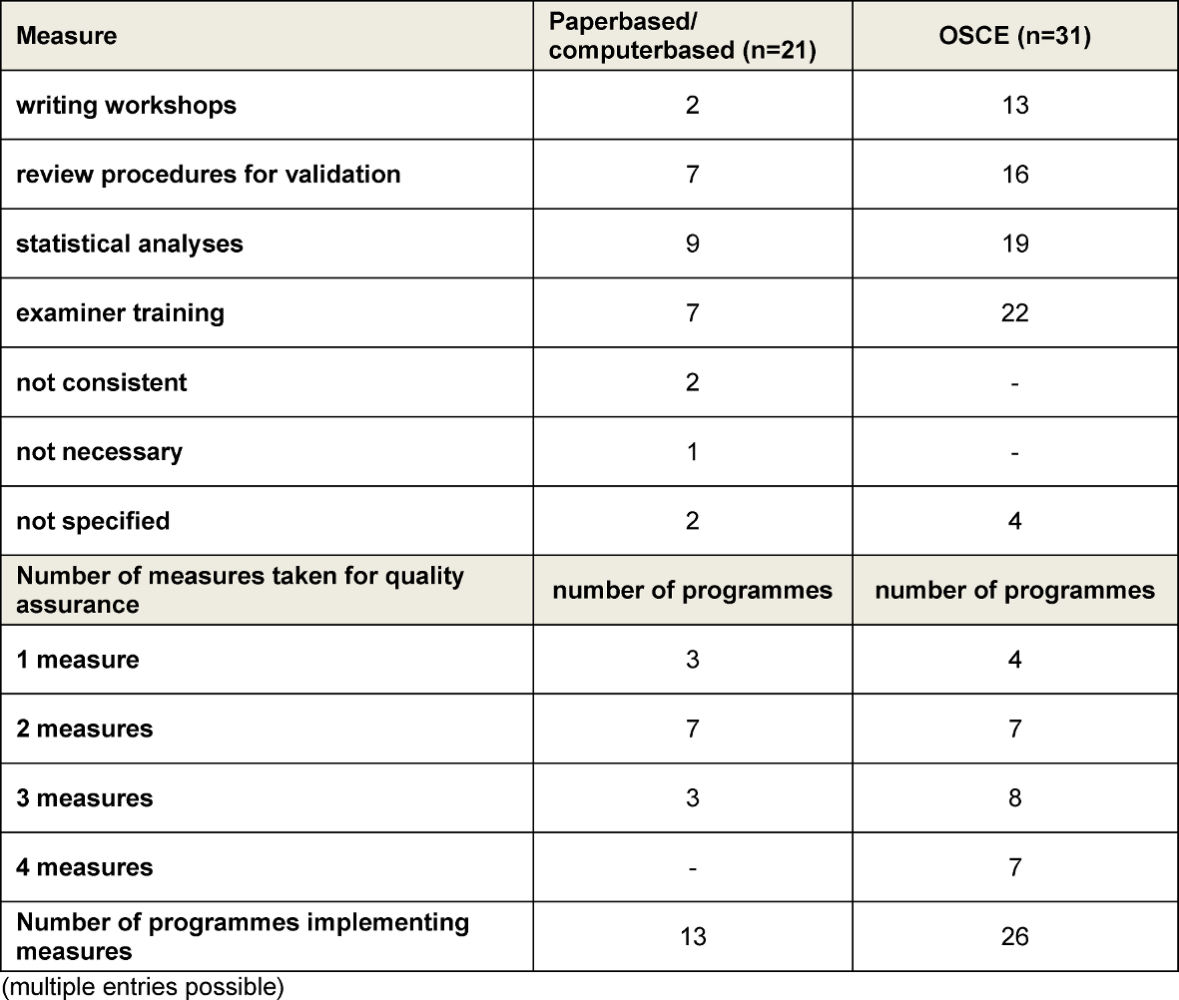
Implemented methods for the quality assurance of assessments, as well as the number of methods per programme
