# Supplementary material for: Desire and reality – teaching and assessing communicative competencies in undergraduate medical education in German-speaking Europe – a survey
Source: GMS Z Med Ausbild. 2015 Nov 16;32(5):Doc56. doi: 10.3205/zma000998 (PMC4647163; doi:10.3205/zma000998)
Supplement: The following is the survey questionnaire for communicative competencies in medical studies in German-speaking Europe. [file ZMA-32-56-s-001.pdf]

Attachment 1

The following is the survey questionnaire for communicative competencies in medical studies in German-speaking Europe. Not all questions were asked automatically. Many of the questions only appeared after a certain answer was chosen in a preceding question.

1. Site

1. Country:
2. City / Location
3. University name / Faculty name
4. Degree programme name
5. How many students complete your degree programme annually?

2. Description of curriculum

1. How are communicative competencies instructed in your programme?
2. How familiar are you with the curriculum of your programme?
3. In which academic years are communicative competencies instructed?
4. Are the cross-semester courses for the transmission of communicative and social competencies coordinated (e.g., in the sense of a longitudinal curriculum)?
5. Is/are the curriculum/the course/the courses oriented on a superordinate model/learning objectives catalogue?
6. Are specific techniques, schemes or the like conveyed?
7. What are the sizes of the groups in which communicative and social competencies are instructed?
8. Which pedagogical elements are used?
9. In which circumstances do the students receive feedback?
10. Who delivers the feedback?
11. What do the students receive feedback on?
12. Are observation forms used? If so, what type?
13. Which theories or models provide the basis for feedback (e.g., theme-centred interaction?)
14. Comments/additional information describing your programme

3. <u>Description of the assessment system</u>

1. Are communicative and social competencies assessed with relevance to passing (summative) in your programme?
2. How familiar are you with the assessments in your programme?
3. In which years of study are communicative and social competencies assessed with relevance to passing?
4. Are scores given in the scope of pass-relevant assessments for communicative and social competencies?
5. Are communicative and social competencies assessed without relevance to passing (formative) in your programme?
6. In which years of study are communicative and social competencies assessed without relevance to passing?
7. Which assessment formats are used in general (summative and formative) in the assessment of communicative and social competencies?
8. Is there a central examination department for support in the preparation, implementation and/or evaluation of assessments?

4. <u>Paper-based and computer-based assessment</u>

1. In which years of study do paper-based or computer-based assessments take place?
2. Are communicative and social competencies evaluated in paper-based or computer-based pass-relevant individual assessments?
3. Are communicative and social competencies evaluated in pass-relevant paper-based or computer-based combined assessments (in combination with other formats, competencies, knowledge)?
4. Which question formats or scenarios that prompt students to an answer (stimuli) are used?
5. Which response formats are used?
6. How is the cut-off score for assessments determined?
7. In what form do students receive feedback?
8. Who is responsible for the evaluation of the assessments?
9. Which disciplines do the examiners come from (e.g., medical psychology, internal medicine …)?
10. Are the examiners also part of the teaching staff?

11. Which of the following measures for quality assurance of the paper-based/computer-based assessments are implemented in your programme?
12. Please provide a brief description of the type of examiner training including the length of training.
13. Comments/additional information on paper-based and/or computer-based assessments

5. <u>Objectified structured examinations</u>

1. How many OSCEs are there in your programme in total?
2. In how many OSCEs are communicative and social competencies assessed in your programme?
3. In which years of study are communicative and social competencies assessed by means of an OSCE?
4. The OSCEs are composed of the stations …
5. Which instruments are used for evaluation for communicative and social competencies in the OSCE in your programme?
6. Which validated instruments are used in your programme?
7. What type of evaluation instrument for communicative and social competencies in the OSCE is used in your programme?
8. How is the cut-off score in OSCEs determined in your programme?
9. What form of feedback do students receive?
10. Who functions as examiner in an OSCE for communicative and social competencies?
11. Which disciplines are the physicians from (e.g., medical psychology, internal medicine, various)?
12. Do the examiners also teach communicative and social competencies?
13. Which of the measures for quality assurance of the OSCEs are implemented in your programme?
14. Please provide a short description of the type of training that the OSCE examiners complete including the length of training.
15. Comments/additional information on OSCEs

6. Workplace-based assessment

16. In which years of study does a workplace-based assessment (WBA) take place for communicative and social competencies?
17. Which competencies are assessed in the scope of the WBA?
18. Which instruments are used in your programme for the evaluation of communicative and social competencies?
19. Which validated instruments are used in your programme?
20. Is the WBA relevant to passing?
21. Comments/additional information on the WBA

7. Portfolio

22. In which years of studies are evaluations/assessments carried out for the portfolio?
23. Which competencies are assessed in the scope of the portfolio?
24. How many components/assessments are in the portfolio for communicative and social competencies?
25. Who functions as examiner for the portfolio?
26. Is the portfolio assessment for communicative and social competencies relevant to passing or part of a pass-relevant assessment?
27. Comments/additional information on the portfolio

8. Comments and additional information on the survey

28. If you found that any questions were lacking or unclear, please share this with us here:
29. Please share any comments/suggestions/additional information here:
30. Who is the contact person for queries regarding this survey? (name, telephone number and e-mail, if possible)
