# Supplementary material for: Desire and reality – teaching and assessing communicative competencies in undergraduate medical education in German-speaking Europe – a survey
Source: GMS Z Med Ausbild. 2015 Nov 16;32(5):Doc56. doi: 10.3205/zma000998 (PMC4647163; doi:10.3205/zma000998)
Supplement: Data on the years of study in which communicative competencies are instructed in the curricula and whether a longitudinal communication curriculum has been implemented in full, in part or not at all (academic year=AJ) [file ZMA-32-56-s-002.pdf]

Attachment 2: Data on the years of study in which communicative competencies are instructed in the curricula and whether a longitudinal communication curriculum has been implemented in full, in part or not at all (academic year = AJ)

**Due to the modular design of the curriculum, allocation to academic years not entirely possible.*

No.	Longitudinal communication curriculum implemented	1st AY	2nd AY	3rd AY	4th AY	5th AY	6th AY	AY total
**1**	Yes	Yes	Yes	Yes	Yes	Yes	Yes	6
**2**	Yes	Yes	Yes	Yes	Yes	Yes	Yes	6
**3**	Yes	Yes	Yes	Yes	Yes	Yes	No	5
**4**	Yes	Yes	Yes	Yes	Yes	Yes	No	5
**5**	Yes*	Yes	Yes	No	Yes	Yes	Yes	5
**6**	Yes	Yes	Yes	Yes	Yes	No	No	4
**7**	Yes	No	Yes	Yes	Yes	Yes	No	4
**8**	Yes	Yes	No	Yes	No	No	Yes	3
**9**	Yes	No	No	Yes	Yes	Yes	No	3
**10**	Yes	Yes	Yes	No	No	No	No	2
**Sum 1-10:**	**10**	**8**	**8**	**8**	**8**	**7**	**4**	
**11**	In part	Yes	Yes	Yes	Yes	Yes	Yes	6
**12**	In part	Yes	Yes	Yes	Yes	Yes	Yes	6
**13**	In part	Yes	Yes	Yes	Yes	Yes	Yes	6
**14**	In part	Yes	Yes	Yes	Yes	Yes	Yes	6
**15**	In part	Yes	Yes	Yes	Yes	Yes	Yes	6
**16**	In part	Yes	Yes	Yes	Yes	Yes	Yes	6
**17**	In part	Yes	Yes	Yes	Yes	Yes	Yes	6
**18**	In part	Yes	Yes	Yes	Yes	Yes	No	5
**19**	In part	Yes	Yes	Yes	Yes	Yes	No	5
**20**	In part	Yes	Yes	Yes	Yes	Yes	No	5
**21**	In part	Yes	Yes	Yes	Yes	Yes	No	5
**22**	In part	Yes	Yes	Yes	Yes	Yes	No	5
**23**	In part	Yes	Yes	Yes	Yes	No	No	4
**24**	In part	Yes	Yes	Yes	No	Yes	No	4
**25**	In part	No	Yes	Yes	No	Yes	Yes	4
**26**	In part*	Yes	Yes	Yes	Yes	No	No	4
**27**	In part	No	Yes	Yes	Yes	Yes	No	4
**28**	In part	No	Yes	Yes	Yes	No	Yes	4
**29**	In part	No	Yes	Yes	Yes	Yes	No	4
**30**	In part	Yes	No	Yes	Yes	No	No	3
**31**	In part	Yes	Yes	Yes	No	No	No	3
**32**	In part	Yes	No	No	Yes	No	Yes	3
**33**	In part	Yes	Yes	No	No	No	No	2
**34**	In part	No	Yes	Yes	No	No	No	2
**35**	In part*	No	No	No	No	No	No	0
**Sum 11-35**	**25**	**19**	**22**	**22**	**19**	**16**	**10**	
**36**	No	No	Yes	Yes	Yes	Yes	Yes	5
**37**	No	Yes	No	Yes	Yes	Yes	No	4
**38**	No	No	Yes	Yes	Yes	Yes	No	4
**39**	No	Yes	No	Yes	Yes	No	No	3
**40**	No	No	Yes	No	No	No	No	1
**Sum 36-40**	**5**	**2**	**3**	**4**	**4**	**3**	**1**	
**Sum 1-40**	**40**	**39**	**33**	**34**	**31**	**26**	**15**	
