# Supplementary material for: Desire and reality – teaching and assessing communicative competencies in undergraduate medical education in German-speaking Europe – a survey
Source: GMS Z Med Ausbild. 2015 Nov 16;32(5):Doc56. doi: 10.3205/zma000998 (PMC4647163; doi:10.3205/zma000998)
Supplement: Evaluations timeframe for summative (pass-relevant; “s”) assessments and formative (not pass-relevant; ”f”) assessments over the years of study [file ZMA-32-56-s-003.pdf]

Attachment 3: Evaluations timeframe for summative (pass-relevant; “s”) assessments and formative (not pass-relevant; ”f”) assessments over the years of study

Nr.	summative	formative	1st AY s	1st AY f	2nd AY s	2nd AY f	3rd AY s	3rd AY f	4th AY s	4th AY f	5th AY s	5th AY f	6th AY s	6th AY f	summative sum	formative sum	total (s&f)
1	Yes	unknown	No	No	No	No	Yes	No	Yes	No	Yes	No	No	No	3	0	3
2	Yes	unknown	No	No	Yes	No	No	No	No	No	Yes	No	No	No	2	0	2
3	Yes	unknown	No	No	Yes	No	No	No	No	No	No	No	No	No	1	0	1
4	Yes	unknown	No	No	No	No	No	No	Yes	No	No	No	No	No	1	0	1
5	Yes	unknown	No	No	No	No	No	No	No	No	No	No	No	No	0	0	0
6	Yes	Yes	Yes	No	Yes	No	Yes	No	Yes	No	Yes	No	No	Yes	5	1	6
7	Yes	Yes	No	No	Yes	Yes	No	Yes	Yes	Yes	No	Yes	No	No	2	4	6
8	Yes	Yes	No	No	No	No	Yes	No	No	No	Yes	Yes	Yes	Yes	3	2	5
9	Yes	Yes	No	No	No	Yes	Yes	No	Yes	No	Yes	No	No	No	3	1	4
10	Yes	Yes	No	No	No	No	No	No	Yes	Yes	No	Yes	Yes	No	2	2	4
11	Yes	Yes	Yes	No	No	Yes	No	No	No	Yes	No	No	No	Yes	1	3	4
12	Yes	Yes	No	No	No	No	No	Yes	Yes	No	Yes	No	No	No	2	1	3
13	Yes	Yes	No	No	Yes	Yes	No	No	Yes	No	No	No	No	No	2	1	3
14	Yes	Yes	No	No	Yes	No	No	Yes	No	No	Yes	No	No	No	2	1	3
15	Yes	Yes	No	Yes	No	Yes	No	No	No	No	Yes	Yes	No	No	1	2	3
16	Yes	Yes	No	No	No	No	No	Yes	No	Yes	No	Yes	No	No	0	3	3
17	Yes	Yes	No	No	No	No	Yes	No	No	No	No	Yes	No	No	1	1	2
18	Yes	Yes	No	No	No	No	Yes	No	No	No	No	No	No	Yes	1	1	2
19	Yes	Yes	No	No	Yes	No	No	No	No	Yes	No	No	No	No	1	1	2
20	Yes	Yes	No	No	No	No	No	No	Yes	Yes	No	No	No	No	1	1	2
21	Yes	Yes	No	No	Yes	No	No	No	No	Yes	No	No	No	No	1	1	2
22	Yes	No	Yes	No	Yes	No	No	No	Yes	No	Yes	No	No	No	4	0	4
23	Yes	No	No	No	No	No	Yes	No	Yes	No	Yes	No	Yes	No	4	0	4
24	Yes	No	No	No	No	No	Yes	No	Yes	No	Yes	No	No	No	3	0	3
25	Yes	No	No	No	No	No	Yes	No	No	No	Yes	No	No	No	2	0	2
26	Yes	No	Yes	No	Yes	No	No	No	No	No	No	No	No	No	2	0	2
27	Yes	No	No	No	Yes	No	No	No	No	No	Yes	No	No	No	2	0	2
28	Yes	No	No	No	No	No	Yes	No	Yes	No	No	No	No	No	2	0	2
29	Yes	No	No	No	No	No	Yes	No	Yes	No	No	No	No	No	2	0	2
30	Yes	No	No	No	No	No	Yes	No	No	No	No	No	No	No	1	0	1
31	Yes	No	No	No	Yes	No	No	No	No	No	No	No	No	No	1	0	1
32	Yes	No	No	No	No	No	No	No	No	No	No	No	No	No	0	0	0
33	No	Yes	No	Yes	No	Yes	No	Yes	No	No	No	Yes	No	No	0	4	4
34	No	Yes	No	Yes	No	No	No	No	No	Yes	No	No	No	Yes	0	3	3
35	No	Yes	No	No	No	Yes	No	Yes	No	No	No	No	No	Yes	0	3	3
36	No	No	No	No	No	No	No	No	No	No	No	No	No	No	0	0	0
37	No	No	No	No	No	No	No	No	No	No	No	No	No	No	0	0	0
38	No	No	No	No	No	No	No	No	No	No	No	No	No	No	0	0	0
39	No	No	No	No	No	No	No	No	No	No	No	No	No	No	0	0	0
40	No	No	No	No	No	No	No	No	No	No	No	No	No	No	0	0	0
		Sum Nos. 1-40	4	3	12	7	12	6	14	8	13	7	3	6			
